# The effect of directional social cues on saccadic eye movements in Parkinson’s disease

**DOI:** 10.1007/s00221-021-06034-7

**Published:** 2021-04-29

**Authors:** Koray Koçoğlu, Gülden Akdal, Berril Dönmez Çolakoğlu, Raif Çakmur, Jagdish C. Sharma, Gemma Ezard, Frouke Hermens, Timothy L. Hodgson

**Affiliations:** 1 Department of Neurosciences, Institute of Health Sciences, Dokuz Eylül University, Izmir, Turkey; 2 Department of Neurology, Faculty of Medicine, Dokuz Eylül University, Izmir, Turkey; 3 Lincoln County Hospital, Lincoln, UK; 4 School of Psychology, University of Lincoln, Lincoln, LN6 7TS UK

**Keywords:** Attention, Saccades, Parkinson’s disease

## Abstract

There is growing interest in how social processes and behaviour might be affected in Parkinson’s disease. A task which has been widely used to assess how people orient attention in response to social cues is the spatial cueing task. Socially relevant directional cues, such as a picture of someone gazing or pointing to the left or the right have been shown to cause orienting of visual attention in the cued direction. The basal ganglia may play a role in responding to such directional cues, but no studies to date have examined whether similar social cueing effects are seen in people with Parkinson’s disease. In this study, patients and healthy controls completed a prosaccade (Experiment 1) and an antisaccade task (Experiment 2) in which the target was preceded by arrow, eye gaze or pointing finger cues. Patients showed increased errors and response times for antisaccades but not prosaccades. Healthy participants made most anticipatory errors on pointing finger cue trials, but Parkinson's patients were equally affected by arrow, eye gaze and pointing cues. It is concluded that Parkinson's patients have a reduced ability to suppress responding to directional cues, but this effect is not specific to social cues.

## Introduction

Parkinson’s disease (PD) is a progressive neurodegenerative disorder with insidious onset (Kalia and Lang 2015). Neuropathologically, PD is most clearly characterized by a loss of dopaminergic cells in the substantia nigra pars compacta (SNc) (Shulman et al. 2011) which send projections to the striatum (caudate and putamen), which itself is functionally connected with overlying regions of the frontal cerebral cortex via cortico-striatal loops (Alexander and Crutcher 1990). Whilst its most obvious symptoms are motoric (e.g., slowness, stiffness and shaking), non-motor symptoms including cognitive deficits are well documented (Aarsland et al. 2010; Lanciego et al. 2012; Hu et al. 2011). There is also increasing interest in social processing deficits in PD, in particular problems affecting social perception. For example, a number of recent studies have shown impaired emotion recognition in PD (Coundouris et al. 2019; Henry et al. 2015; Pohl et al. 2017), perhaps due to depletion of dopamine within fronto-striatal circuits mediating affect and emotion. An aspect of social perception in Parkinsons that has not been investigated to date is the orienting of attention in response to social cues. In this paper we describe performance of patients with Parkinson’s disease (PDs) in an eye movement task in which directional cues are presented at fixation, whilst peripheral saccade targets appear to the left or right. The responses of patients to targets preceded by social or non-social cues were compared to that of an age matched control group.

A common test used to investigate orienting of attention is the spatial cueing paradigm, in which the onset of a peripheral target for a motor response or decision is preceded by a directional cue such as an arrow presented at central fixation (Posner and Cohen 1984; Posner et al. 1978, 1985). A number of studies have found that the difference in manual reaction times to targets appearing in locations congruent or incongruent with the direction of a centrally presented arrow is reduced in PDs, suggesting reduced allocation of attention or response readiness towards stimuli appearing at the cued location in patients (Bennett et al. 1995; Filoteo et al. 1997; Hsieh et al. 1996; Pollux and Robertson 2001; Wright et al. 1990; Yamaguchi and Kobayashi 1998; Yamada et al. 1990). Orienting in response to exogenous cues, such as a peripheral onset preceding the target stimulus, is also affected in PD. For example, Briand et al. (2001) found that the facilitatory effect of exogenous attentional cues on saccades to peripheral targets is reduced in advanced but not mildly effected patients.

Other work has examined how PD affects responses to natural objects which have a strong action “affordance” (see Gibson 1979). Poliakoff et al. (2007) and Galpin et al. (2011) asked participants to judge the colour of an object (green or blue) presented on a computer screen, using a left or right key press. The object was either a picture of a door handle or an abstract image of a meaningless object with similar visual and orientation properties to the door handle. The direction in which the object was oriented (left or right) exerted a response compatibility effect, such that left key presses were quicker in response to a leftward oriented object and vice versa for rightward responses. Healthy participants showed a larger compatibility effect for images of door handles, suggesting the existence of an object action affordance influencing responding. Although patients demonstrated spatial compatibility effects, they did not show an additional action affordance effect for door handles, implying that internal activation of actions by external cues may be disrupted in PD.

Many studies have shown that briefly displaying a picture of a face or eyes looking to the left or right facilitates visuospatial attention and overt saccadic eye movements in the direction in which the eyes in the cue image are gazing. This eye gaze cuing effect is found even when participants are instructed to ignore the direction in which the eyes point and when cues are uninformative with respect to the likely direction in which the target object appears (Driver et al. 1999; Friesen and Kingstone 1998; Gregory and Hodgson 2012; Koval et al. 2005; Kuhn and Benson 2007; Kuhn and Kingstone 2009; Kuhn et al. 2009). It has been suggested that eye gaze cueing effects are reflective of fundamental mechanisms underpinning social interaction and “mind-reading” abilities in humans (Baron-Cohen 1994). Specifically, the obligatory or "reflexive" nature of orienting in response to gaze cues may constitute evidence for the existence of an innate, hard-wired eye gaze direction detector module within the brain (Baron-Cohen 1995). Social cues might also uniquely activate associated action affordances (Gibson 1979), causing a “mirroring” of the observed orienting response in the viewer (De Bordes et al. 2019). An alternative perspective that could explain the effect is that responses initiated by eye gaze and other directional social cues are not hard-wired in the traditional sense, but are acquired via repeated pairing of stimuli in the environment with orienting of attention, i.e., learned stimulus–response (SR) associations. Consistent with this idea, uninformative non-biological stimuli (arrows) have in fact been shown to produce very similar obligatory cueing effects to gaze cues (Galfano et al. 2012; Kuhn and Benson 2007; Kuhn and Kingstone 2009; Quadflieg et al. 2004; Ristic et al. 2002; Tipples 2002, 2008).

In this study we asked people with Parkinson’s disease and age-matched healthy control participants to complete a prosaccade task in which the onset of a target for an eye movement was preceded by a directional cue depicting either an image of a pair of eyes, a pointing hand or an arrow pointing to the left or right. The cue was equally likely to point either in a congruent (same) or incongruent (opposite) direction to the saccade target. For prosaccades, normal amplitude and response latency are typically reported in PD (Briand et al. 1999; Fukushima et al. 1994; Hodgson et al. 1999; Lueck et al. 1990; Mosimann et al. 2005). Therefore, whilst we expected eye movements to be of normal amplitude and reaction time overall, we were interested in whether PDs differed from controls with respect to the magnitude of cue-congruency effects. If socially derived cueing effects relied on the same mechanism as other attentional cueing effects, then differences might be expected between controls and patients in the task regardless of cue type. However, if social cues relied on different pathways then a selective preservation or impairment of the effect of social cues (eyes/pointing) relative to non-socially relevant cues (arrows) might be expected in patients.

## Experiment 1

### Methods

#### Participants

Participants were recruited via a movement disorders clinic at the Lincoln County Hospital, UK, the Parkinson’s UK research network as well as the outpatient clinic in the Department of Neurology at Dokuz Eylül University Hospital, Izmir, Turkey. Older controls were spouses, close relatives or friends of the patients at both recruitment sites. Twenty-three patients with mild to moderately severe Parkinson’s disease (13 males, 10 females, mean age 66.48 ± 7.73 years) and 13 healthy controls (7 males, 6 females, mean age 63.92 ± 5.58 years) took part in the study. Fifteen patients and 10 controls were recruited from Izmir with 8 patients and 3 older controls coming from the UK. Symptom severity was assessed using the United Parkinson's Disease Rating Scale III (UPDRS III motor score) (Fahn and Elton 1987). General cognitive function was assessed in patients and controls using the Mini Mental State Examination (MMSE) (Folstein et al. 1975). Eighteen out of the 23 patients and 4 out of the 13 controls completed several other neuropsychological tests at the testing session including forward and backward digit span, Stroop test and verbal fluency assessments. Patients were mild to moderately affected with a mean UPDRS III of 15.48 ranging from 4 to 33. They were also found as a group to have lower MMSE scores than the control group, although this difference was not statistically significant and all patients scored 23 or over on the test, i.e., none of the scores were low enough to support a diagnosis of dementia (Mean MMSE Controls: 29.00 ± 0.51; PDs: 27.84 ± 0.4).

Participants provided written informed consent to participate in the study. All participants had normal or corrected to normal vision. Controls had no history of neurological and psychiatric problems. The study was approved by the local Ethics Committees for the School of Psychology, University of Lincoln and NHS regional ethics committee (UK) and the ethics committee of the Dokuz Eylül University Hospital (Turkey), in accordance to the principles of the Declaration of Helsinki.

#### Apparatus and stimuli

**Fig. 1:**
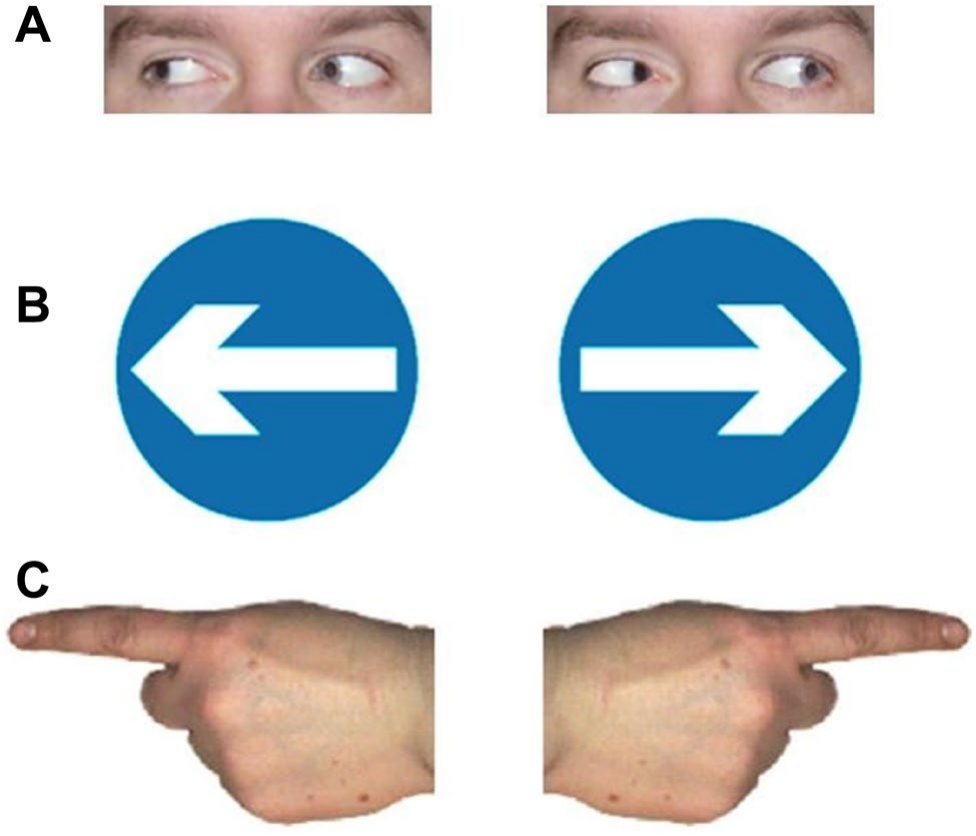
Cue stimuli used in Experiments 1 and 2. **a** Eye gaze cues. **b** Arrow cues. **c** Pointing finger cues. The cue appeared prior to appearance of the saccade target to the left or right on each trial and was equally likely to point in either a direction congruent or incongruent with respect to the target direction

Subjects were tested using an Eyelink 1000 (Lincoln) or EyeLink 1000 Plus (Izmir) eye-tracker (SR Research Ltd., Ontario, Canada). The viewing distance to the monitor was approximately 60 cm. Stimuli were presented on a 19 inch LCD monitor (60 Hz frequency; 1024 × 768 screen resolution) attached to a Dell PC. The pupil movement was tracked using infrared camera with a frequency of 1000 Hz. A chin rest was used to keep the head in a stable position. Corel Paint Shop Pro X image editing software was used to create the cue stimuli used. The eye gaze images were colour photographs of a Caucasian male face cropped to show only the eyes, subtending 5.52° of visual angle. The arrow cues were based on the left and right road directional signage used in the UK, Europe and Turkey, comprising a blue circle with a white arrow. The arrow cues items subtended a visual angle of 4.45°. The finger pointing stimuli consisted of colour image of a whole male hand, with a pointing indexing finger subtending 5.52° visual angle (Fig. 1).

#### Procedure

A modified prosaccade task was used in which a peripheral target onset was preceded by presentation of one of the 6 central cues depicting either a pointing finger, eyes or arrows directed towards the left or right side of the screen. The 6 different cue types and directions occurred with equal likelihood in a randomised order within a single block of 48 trials, preceded by presentation of 6 practice trials in which each cue type was included once. The task length was kept as short as possible to reduce participant discomfort and to reduce overall length of the testing session, which also included other assessments and eye movement tests. Collapsing across cue and target direction there were 4 trial repeats per cue type, congruency and SOA condition for each participant, or 8 trials per condition if collapsing across SOA.

**Fig. 2:**
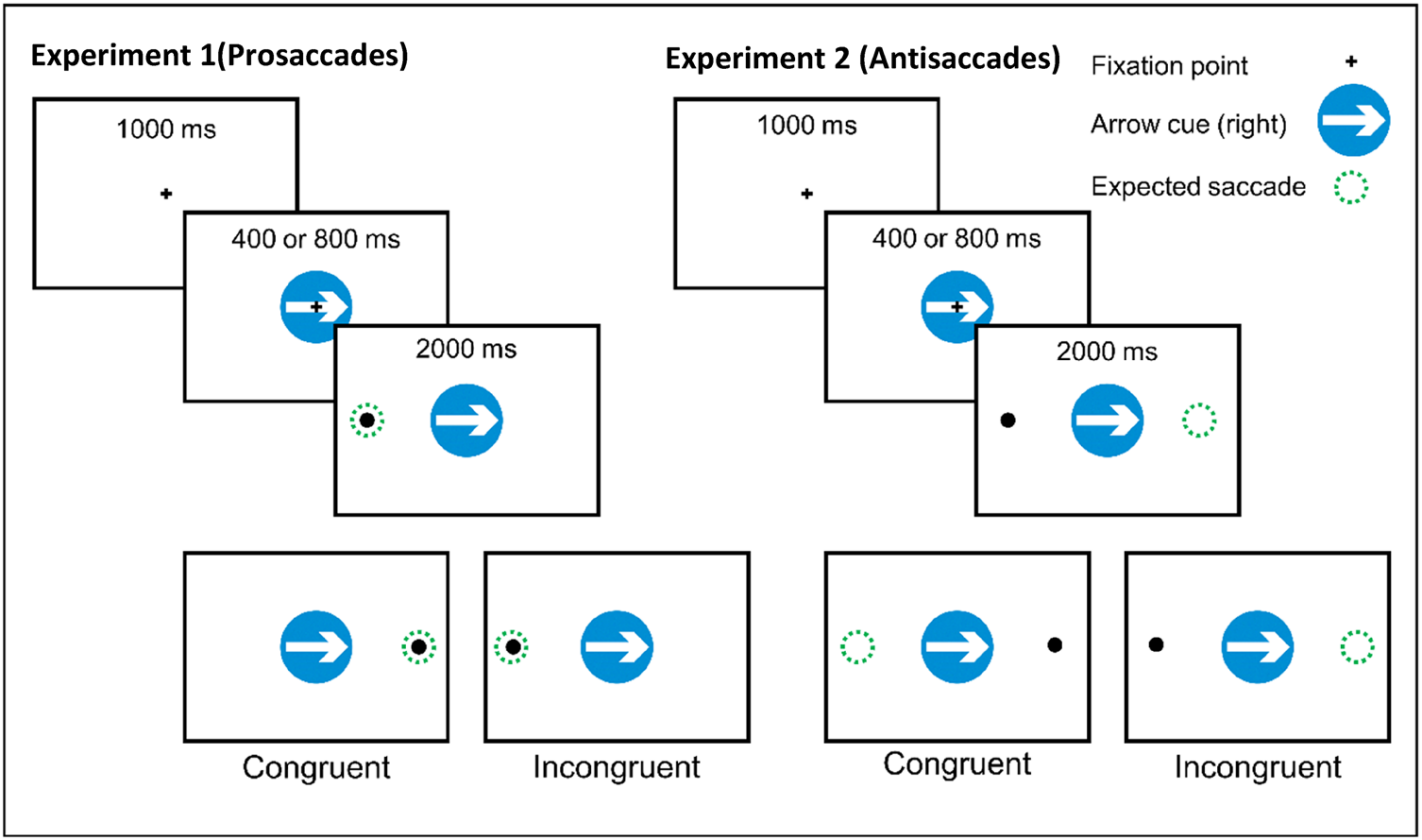
Schematic illustrating cued saccade task, with trials using arrows as the pre-cue image shown. The green circle indicates the correct location for the saccade response on the two example trials and for congruent and incongruent trial types in each case

Each individual trial in the task began with the presentation of a black central fixation cross subtending 0.95 degrees of visual angle, for a duration of 1000 ms. Following this, the cue was presented at fixation. After a stimulus onset asynchrony (SOA) of either 100 or 500 ms, the central fixation cross was extinguished and a black dot with a diameter of 1.26° of visual angle. The dot could appear vertically centred at either the left or right of the screen at an eccentricity of 14.75° of visual angle, where it remained for 2000 ms after which both the target and cue were extinguished. The target dot appeared on the left and right sides of the screen with equal probability within a block. Participants were instructed to “maintain fixation on the central cross and ignore the road signs, fingers and eyes until the dot appeared, when they should look at the dot with their eyes". Participants were also informed that the direction to which the arrows or eyes pointed did not predict the likely position of the target dot. An interval of 1000 ms separated the beginning of one trial and the start of the next. A schematic of the events on a prosaccade task trial is shown in Fig. 2.

#### Data analysis

Saccade parameters were extracted off-line with Dataviewer software (SR Research, Canada). Saccades were detected as those eye movements which exceeded a velocity of 30°/s and an acceleration of 8000°/s/s. The first saccade detected by Dataviewer following cue onset was taken as the response for each trial for each participant and only those which occurred between 80 and 1500 ms (see Fischer et al. 1997) from target onset were included in the analysis of saccade response times (SRTs) and saccades which were initiated earlier than 80 ms before the target onset and exceeded an amplitude of 2.0° were classified as anticipatory movements. Of the remaining saccades, those which were initiated in the same direction as the peripheral stimulus were considered as correct responses, whilst those made in the opposite direction of the peripheral stimulus were classed as direction errors. Across all participants tested and trials analysed a total of 53 trials also had to be excluded due to data loss (either due to eye-lid interference or poor eye tracker calibration), with a maximum of 5 trials excluded in a single task block in the case of one of the participants.

Analysis of Variance (ANOVA), *t* tests and Pearson correlations were initially carried out using SPSS 25 (IBM inc.). Equivalent Bayesian analyses were completed using JASP (Version 0.13.1) software to calculate *inclusion Bayes Factors* (BF_incl_). For each main effect and interaction effect the BF_incl_ provides an indication of how much more likely models which include the effect are, relative to the average likelihood of all models that do not include the effect (relative to the null hypothesis model). In the case of interaction effects, the likelihood comparison is made between models that include the interaction and those which include at least one of the component effects of the interaction as main effects or in lower order interactions (the “across matched models” option in JASP). Although there are no hard boundaries for considering the statistical significance of Bayes Factors, values between 0.33 and 3 are generally considered to offer weak evidence for distinguishing between the modelled and null hypotheses, whereas values greater than 3 or less than 0.33 are considered strong evidence for or against the tested model.

### Results

#### Saccade reaction time

**Fig. 3:**
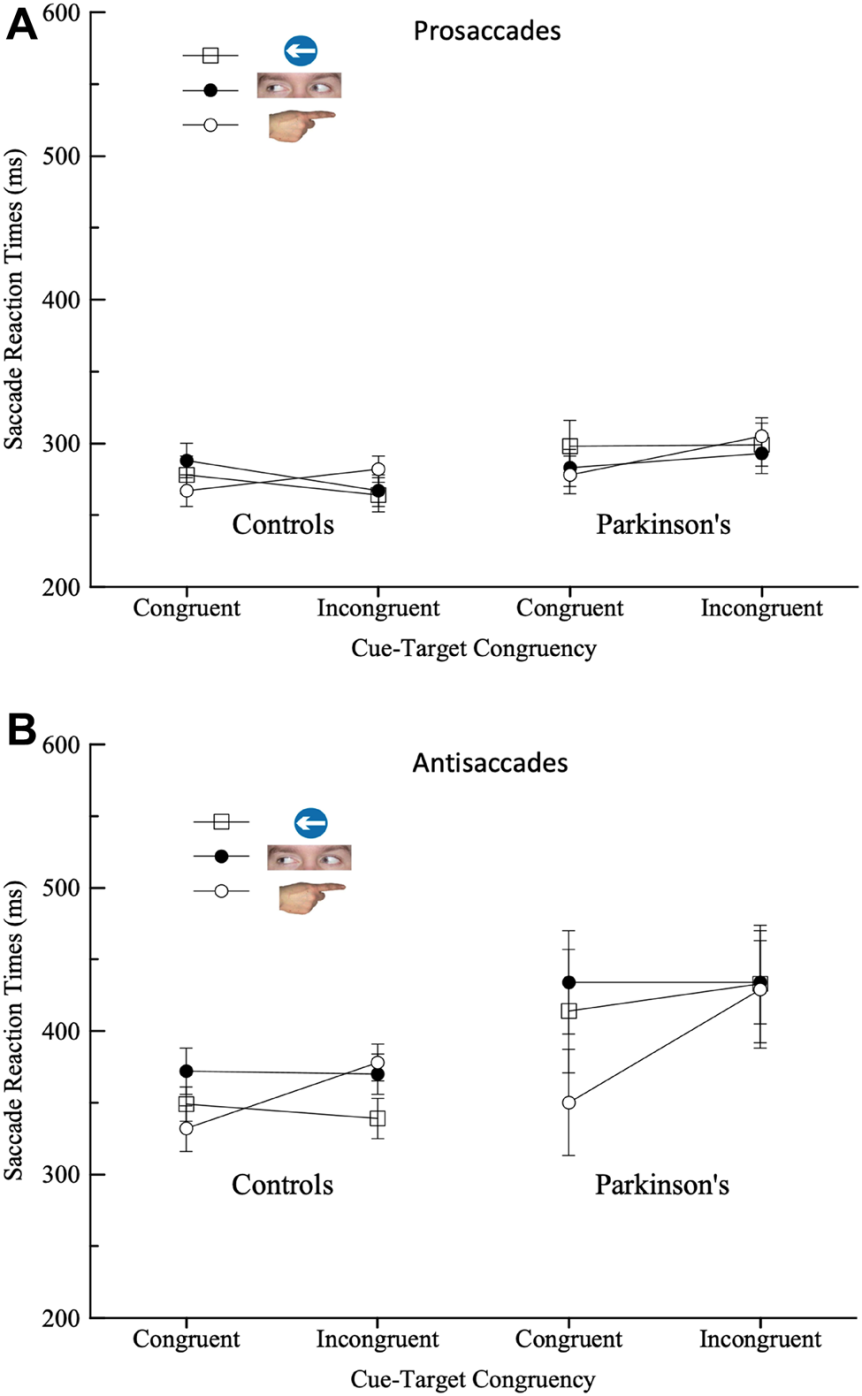
Saccade response times by cue-target direction congruency for the three cue types, for patients and control participants in **a** Experiment 1 (prosaccades) and **b** Experiment 2 (antisaccades)

Due to low trial numbers and high rates of directional and anticipatory errors (see below), saccadic reaction times (SRTs) were pooled across trials with different SOAs (100/500 ms) to obtain a mean SRT value for each participant in each of the cue type and congruency conditions. A three-way mixed Analysis of Variance (ANOVA) with 2 repeated measures factors: cue type (Arrows; Eyes; Fingers), cue-target direction congruency (congruent; incongruent) and one between participants factor: Group (Patient; Control) showed no main effect of cue type (*F*(2, 62) = 0.483, *p* = 0.483, $\eta_{{\text{P}}}^{2}$ = 0.023; BF_incl_ = 0.07), congruency (*F*(1, 31) = 1.320, *p* = 0.259, $\eta_{{\text{P}}}^{2}$ = 0.041; BF_incl_ = 0.27) or Group (*F*(1, 31) = 0.035, *p* = 0.855, $\eta_{{\text{P}}}^{2}$ = 0.001; BF_incl_ = 0.26). The interaction between cue type and congruency was found to be significant for SRTs (*F*(2, 62) = 3.21, *p* = 0.047, $\eta_{{\text{P}}}^{2}$ = 0.94; BF_incl_ = 0.74). Means comparisons showed that Finger cues were the only condition to show a trend towards a significant congruency effect overall (Eyes: *t*(33) = − 0.268, *p* = 0.884; Arrows *t*(33) = − 0.105, *p* = 0.917; Fingers *t*(33) = 1.80, *p* = 0.081) (Fig. 3a).

#### Saccade direction errors

The proportion of trials on which the primary saccade was directed in the wrong direction (i.e., the first saccade after target onset was made *away* from the target) was analysed using a 4-way Analysis of Variance (ANOVA). This showed a main effect of congruency (*F*(1, 34) = 4.69, *p* = 0.037, $\eta_{{\text{P}}}^{2}$ = 0.12; BF_incl_ = 2.33) and a significant interaction effect between SOA, congruency and Group (*F*(1, 34) = 4.867, *p* = 0.034, $\eta_{{\text{P}}}^{2}$ = 0.13; BF_incl_ = 1.73)**.** Whereas healthy older participants showed a trend towards a congruency effect on directional errors at the shortest SOA (100 ms) (*t*(12) = 1.74, *p* = 0.108) and no difference in errors at the longest (500 ms) SOA, Parkinson's patients showed a significant congruency effect at the longest SOA only (*t*(22) = 2.86, *p* = 0.009) (Table 1).

**Table 1:** Directional saccade errors in the prosaccade task (% of trials) (Experiment 1) for each SOA, congruency condition for the two groups

	SOA/congruency			
	100 ms		500 ms	
	Congruent	Incongruent	Congruent	Incongruent
Controls	1.92 ± 1.48	7.7 ± 2.25	2.75 ± 1.3	2.75 ± 1.7
Parkinson’s	3.6 ± 0.1.12	4.35 ± 1.9	1.8 ± 0.98	5.01 ± 1.28

Whilst the difference in errors between congruent and incongruent trials was greatest at the shortest SOA period for controls, patients show larger cue congruency effect at the longest SOA

The ANOVA also revealed a trend towards an interaction between cue type and congruency which was similar to the interaction for SRTs reported above (*F*(2, 68) = 2.97, *p* = 0.06, $\eta_{{\text{P}}}^{2}$ = 0.08 BF_incl_ = 0.29). Means comparisons revealed a significant cue congruency effect on directional errors for Fingers but not the other cue types (Eyes: *t*(34) = − 0.442, *p* = 0.661; Arrows *t*(35) = 0.442, *p* = 0.661; Fingers *t*(34) = 3.43, *p* = 0.002).

All other main effects and interaction effects for directional errors, including effects of Group, were found to be non-significant at *p* > 0.05.

#### Anticipatory errors

**Fig. 4:**
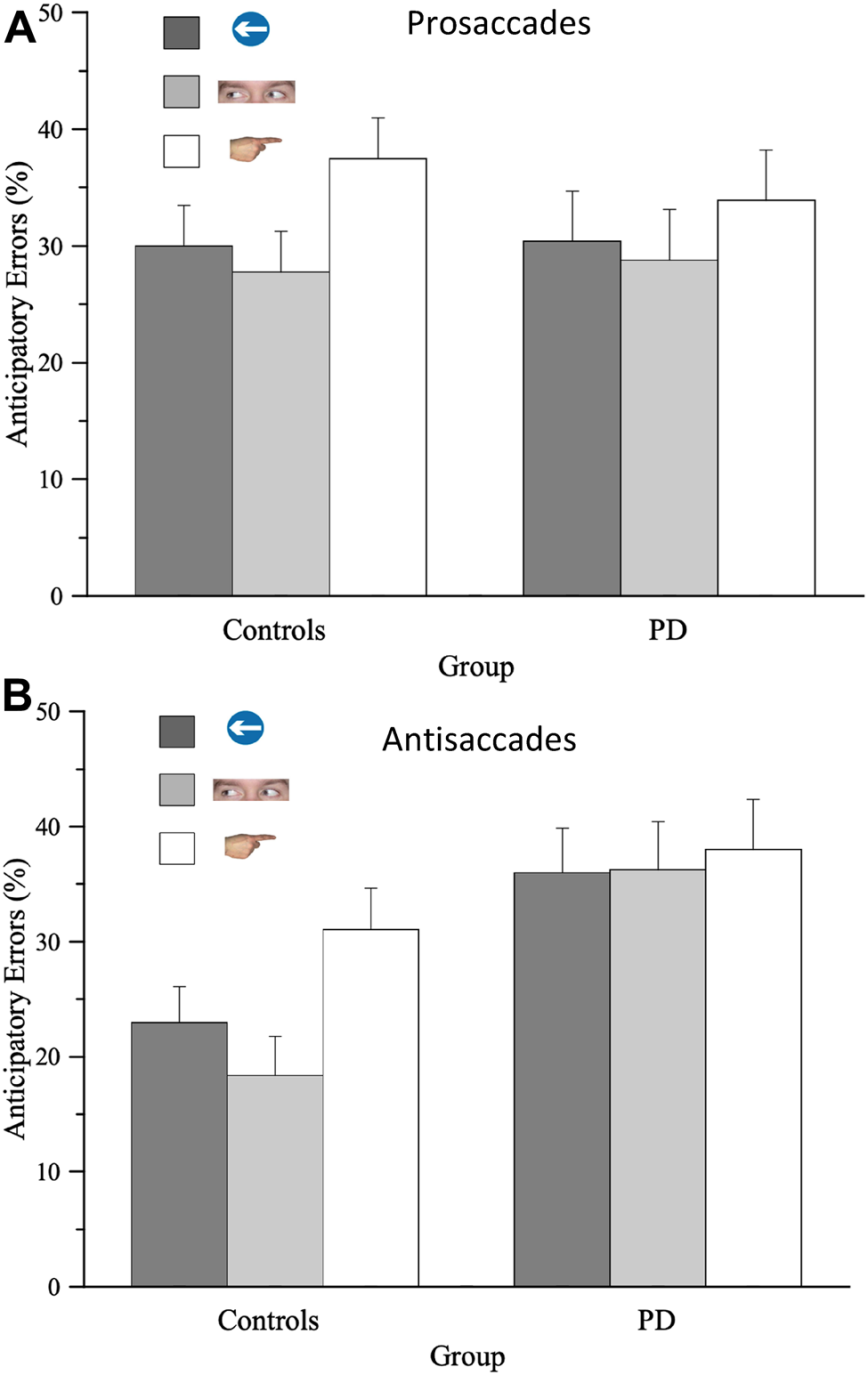
Anticipatory responses in **a** prosaccade task (Experiment 1) and **b** antisaccade task (Experiment 2), under the three different cueing conditions. Whilst Control participants showed the greatest amount of anticipatory responding when pointing finger cues and eye gaze cues preceded the target onset, Parkinsons patients were equally affected by all three cue types

Another analysis looked specifically at anticipatory errors (where a saccade was initiated prior to or within 80 ms of target onset). This showed a significant main effect of SOA (*F*(1, 34) = 70.20, *p* < 0.001, $\eta_{{\text{P}}}^{2}$ = 0.67; BF_incl_ = 1.24exp^27^) with anticipatory errors being increased for trials with a 500 ms versus a 100 ms SOA interval. There was also a strong effect of cue type (*F*(2, 68) = 8.644, *p* < 0.0001, $\eta_{{\text{P}}}^{2}$ = *0.20;* BF_incl=_12.55*),* as well as an interaction between cue type and SOA (*F*(2, 68) = 12.92, *p* < 0.0001; *η*_p_ = 0.03, BF_incl_ = 2739.31). Means comparisons for the size of the effect of SOA on errors showed that this was significantly greater for Finger cues compared to the two other cue types (Fingers versus Eyes: *t*(35) = 5.20, *p* < 0.001; Fingers versus Arrows: *t*(35) = 2.96, *p* = 0.005) (Table 2). A marginally significant interaction effect between Group and cue type was also apparent (*F*(2, 2.49), *p* = 0.069, *η*_p_ = 0.0070, BF_incl_ = 0.62). For Control participants anticipatory errors were increased for Pointing cues relative to both Eye (*t*(12) = 3.36, *p* = 0.006) and Arrow cues (*t*(12) = 2.93, *p* = 0.010), whereas for PDs there were no significant differences between Anticipatory errors for any of the cue types (Fig. 4a). None of the other main effects or interaction effects including those with Group approached significance for the analysis of anticipatory errors.

**Table 2:** Anticipatory responses in the prosaccade task (% of trials) (Experiment 1), for the three different cue types at the two SOA periods

	SOA	
	100 ms	500 ms
Arrows	16.9 ± 3.5	42.8 ± 4.2
Eyes	18.9 ± 3.2	35.8 ± 4.7
Fingers	16.3 ± 3.0	61.5 ± 5.5

Anticipatory errors were significantly increased at longer SOAs and this difference was significantly greater for Finger cues

#### Correlations with Parkinson’s disease severity and cognitive test scores

Pearson correlations were carried out to investigate associations between Parkinson’s disease severity (UPDRS III scores) and cue congruency for each of the three cue types (Arrows, Eyes, Fingers,) for the 3 dependent measures of SRTs, anticipatory errors and directional errors. None of these correlations showed a significant association between UPDRS and cue congruency effects at standard significance level (*α* = 0.05).

Similarly, correlations were carried out between the size of the eye movement cueing effects and scores on cognitive tests for the sub-set of patients completing the neuropsychological tests (see “Methods”) along with the MMSE. These showed correlations between forward and backward digit span and the magnitude of cueing effects on SRTs for Eye Gaze cues (Forward: *ρ* = 0.575, *p* = 0.016; Backward: *ρ* = 0.492, *p* = 0.045); The size of cueing effects on directional errors for Arrow cues and MMSE scores (*ρ* = − 0.427, *p* = 0.048); The size of cueing effects on directional errors for Eye Gaze cues with backward digit span and semantic verbal fluency scores (Digit Span: *ρ* = 0.519, *p* = 0.027; Fluency: *ρ* = 0.661, *p* = 0.037); and between Stroop task interference and anticipatory errors for Arrow cues (*ρ* = − 0.853, *p* = 0.007).

## Discussion (Experiment 1)

As expected there were no significant differences in SRTs overall between patients and controls in Experiment 1, confirming that stimulus driven prosaccades are normal in PD. However, differences were found in how the different cue types (Arrows, Eyes, Fingers) affected saccades. Finger pointing cues showed the greatest cue-congruency effect on SRTs for both PDs and healthy controls, with a trend towards longer SRTs on incongruently cued trials for pointing fingers but not the other cue types (Fig. 3a). Surprisingly, both patients and controls also made errors where a saccade was spontaneously executed in the wrong direction (i.e., *away* from the target) and these errors were found to be more common on cue-target incongruent trials, particularly for pointing finger cues. This suggests that saccade responses were being initiated in response to the cue rather than the target. Consistent with this, rates of anticipatory errors (defined as SRTs < 80 ms) were high overall. Anticipatory errors were particularly common for Pointing cues and and for trials with the longest SOA interval (Table 2). Another interesting difference was found in the time course of the effect of directional cues on errors between PDs and controls. Directional errors were greater for incongruent trials at the 100 ms relative to the 500 ms SOA period for healthy controls but were higher at the 500 ms SOAs for PDs (Table 1).

Previous work has shown cue congruency effects on SRTs for eye gaze cues (Koval et al. 2005), although other work has not found significant effects of eye gaze cues on prosaccades (Gregory 2011). Kuhn et al. (2015) examined overt eye movements in a visual search task, where targets were preceded by dynamic eye gaze cues. Whilst they report gaze cueing effects on saccades in both old and young adults, they found that older adults made fewer overt saccades in the direction indicated by the gaze cue. The present results suggest that eye gaze cues do not produce consistent cue-congruency effects for prosaccades in older adults, whereas pointing finger cues produce more consistent effects. This finding is most reminiscent of results using a very similar cued prosaccade task in children. Gregory et al. (2016) showed that children up to about 7 years showed strong cueing effects for pointing fingers, but congruency effects for eye gaze cues were only apparent in children older than 7 years. The authors suggested that this may reflect the learning and acquisition of stimulus–response associations during early development and that pointing fingers may be amongst the earliest directional cues which children learn to associate with orienting in a particular direction. Rather than socially relevant cues such as eye gaze direction being processed in a privileged way within dedicated brain modules, these findings are more consistent with social cueing effects developing through exposure to consistent patterns of stimulus–response pairings in the environment (e.g., a parent’s pointing finger). It is tempting to speculate that in older age, associations learned earlier in life might re-emerge as dominant mappings as natural aging processes impact on less strongly reinforced stimulus–response pairings. However, larger cueing effects with pointing fingers might equally be explained purely by the physical properties of a pointing hand, which has a more obvious horizontal asymmetry than other cue types (see Fig. 1). Future studies could systematically assess how the visual characteristics of central cues affect visuo-spatial attentional orienting independent of their social or semantic meaning.

The interaction between congruency, SOA and Group seen for directional errors is interesting and implies a difference in the time course for processing directional cues in PDs relative to controls. It suggests that central cues affect saccade generation very quickly in healthy controls (within 100 ms), but this influence then decays (or is actively suppressed) within 500 ms. For Parkinson's patients though, the influence of the central cue seems to persist longer and was greatest for 500 ms SOA trials. This could be due to either a slower decay or a reduced ability to actively inhibit the programming of a saccade in the cue direction in patients. Another way of viewing this is that some of the directional errors at the longest SOA period in patients might be considered “anticipatory” in that they reflect generation of a saccade in the direction of the pre-cue, even though their SRTs were longer than the generally assumed cut-off criteria for deeming a response to be anticipatory.

Apart from this interaction with SOA, the effects of arrows, eye gaze and pointing cues did not differ greatly between patients and controls. From this is might be concluded that the processing of social cues is not differentially affected relative to non-social cues in PD, but this interpretation may be too straightforward. Other work has also found that social processing deficits are only apparent in PDs under conditions where a strongly cued response must be inhibited. For example, Foley et al. (2019) report that Parkinson's patients were only impaired in a version of the classic “Cookie Jar” Theory of Mind test when the inhibitory demands of the test were high. Similarly, differences in the effects of social directional cues between patients and controls might only been seen when the inhibitory demands of an eye movement task are similarly enhanced, placing greater demands on compromised inhibitory control mechanisms in patients. Stimulus-driven saccades might also effectively by-pass cortical centres mediating processing of social cues and the difference in relative influence of social and non-social cues might be expected to be greatest for voluntary movement. Experiment 2 tested this hypothesis using the same task as Experiment 1 but with different participant instructions. This time they were told to refrain from looking at the target stimulus when it appeared to the left or right of the screen and instead make an “antisaccade” (Hallet and Adams 1980) directly towards the screen location opposite the target. In this way the task had an additional response inhibition component to inhibit a stimulus driven saccade towards the peripheral target as well as the requirement to voluntarily generate a saccade towards the location opposite to the target.

## Experiment 2

### Methods

Twenty-one patients with mild to moderately severe Parkinson’s disease (9 males, 12 females, mean age 65.05 ± 9.66 years) and 31 healthy controls (18 males, 13 females, mean age 62.84 ± 6.86 years) participated in Experiment 2. Fifteen patients and 28 controls were recruited from Izmir with 6 patients and 3 older controls coming from Lincoln. Four of the patients and one of the controls had also previously completed the prosaccade task (Experiment 1) either in the same testing session or at a previous testing session. MMSE scores were obtained for all patients and controls and additional neuropsychological testing was carried out in 30 of the control participants and 14 of the patients. MMSE test scores were found to be significantly lower in the patient relative to the control group (means PD: 27.4; controls: 29.4; *t* = 4.11, *df* = 80, *p* < 0.001) although all of the patients scored greater than 23 on the MMSE. Comparisons of digit span, stroop interference and verbal fluency test scores showed no significant differences between groups (digit forward *t*(42) = 0.81, *p* = 0.425; digit backward *t*(42) = 1.13, *p* = 0.267; Stroop *t*(33) = 0.732, *p* = 0.470; fluency *t*(33) = 1.36, *p* = 0.183). All other aspects of the task and procedure were identical to Experiment 1 except for the instruction given to the participant. When the target stimulus appeared following presentation of the central pre-cue (eyes, fingers or arrow), participants were instructed to make a saccade directly away from the target and to fixate the location opposite to that at which the target appeared. A schematic of the events on an antisaccade task trial is shown in Fig. 2.

### Results

#### Saccade reaction time

A three way mixed analysis of variance for correct antisaccade SRTs with cue type, congruency and SOA as repeated measures factors and Group as a between participant factor showed a significant main effect of congruency (*F*(1, 33) = 5.29, *p* = 0.028, $\eta_{{\text{P}}}^{2}$ = *0.14;* BF_incl_ = 0.57) with longer SRTs on incongruent versus congruent trials (i.e., correct antisaccade response times were slower when the cue pointed *away* from the target. Congruent: *M*: 375 ± 11 incongruent: 397 ± 12). A significant main effect of Group was also found with PDs showing significantly slower SRTs in the antisaccade task compared to healthy controls (*F*(1, 33) = 7.435, *p* = 0.010, $\eta_{{\text{P}}}^{2}$ = 0.18; BF_incl_ = 4.55) Parkinson's: *M*: 416 ms ± 19 Controls: *M*: 357 ms ± 10.2). There was also a marginally significant interaction between cue type and congruency (*F*(2, 66) = 2.57, *p* = 0.084, $\eta_{{\text{P}}}^{2}$ = 0.02, BF_incl_ = 2.59). Means comparisons showed a significant congruency effect for finger cues (*t*(38) = 2.72, *p* = 0.01) but no significant effects for eyes (*t*(38) = 0.235, *p* = 0.815) or arrows (*t*(38) = − 0.121, *p* = 0.904) (Fig. 3b). All other factors and interaction effects were found to be non-significant.

#### Saccade direction errors

For non-anticipatory saccade errors, a significant main effect of SOA was found (*F*(1, 50) = 84.80, *p* < 0.001, $\eta_{{\text{P}}}^{2}$ = 0.62; BF_incl_ = 3.53exp^26^) along with a significant main effect of Group (*F*(1, 50) = 10.746, *p* = 0.002, $\eta_{{\text{P}}}^{2}$ = 0.18; BF_incl_ = 14.93), with PDs making more errors relative to controls (Parkinson's M: 25.24% ± 2.56 M: Controls 14.36% ± 2.10). Group did not interact with any other repeated measures factor. However, there was found to be a significant interaction between SOA and congruency (*F*(1, 50) = 6.39, *p* = 0.015, $\eta_{{\text{P}}}^{2}$ = 0.11; BF_incl_ = 1.96) and a 3-way interaction between cue type, SOA and congruency (*F*(2, 100) = 4.08, *p* = 0.020, $\eta_{{\text{P}}}^{2}$ = 0.075; BF_incl_ = 2.08). These interactions reflected a trend towards increased errors on congruent compared to incongruent trials at the short SOA, but significantly increased errors on incongruent compared to congruent trials at the longer 500 ms SOA (*t*(87) = 2.64, *p* = 0.010). This effect of SOA on congruency was strongest for the Arrow and Pointing finger cues but was not apparent for Eye gaze cues (Table 3).

**Table 3:** Effect of cue-target congruency on directional antisaccade errors at different SOAs in Experiment 2

	Cue type/SOA					
	Arrows		Eyes		Fingers	
	100 ms	500 ms	100 ms	500 ms	100 ms	500 ms
Congruent	28.00 ± 3.34	7.02 ± 1.54	28.24 ± 3.48	15.34 ± 2.34	32.28 ± 3.4	7.8 ± 1.92
Incongruent	23.40 ± 2.54	15.00 ± 2.48	29.04 ± 3.14	12.14 ± 2.32	26.52 ± 3.22	12.76 ± 2.46

At longer SOAs, errors were significantly increased on incongruent relative to congruent trials, but this effect was not seen at shorter SOA for eye gaze cues

#### Anticipatory errors

There was a main effect of cue type (*F*(2, 100) = 8.783, *p* < 0.001, $\eta_{{\text{P}}}^{2}$ = 0.15; BF_incl_ = 27.03) and a main effect of SOA (*F*(1, 50) = 98.72, *p* < 0.001, $\eta_{{\text{P}}}^{2}$ = 0.66; BF_incl_ = 1.26exp^46^) on anticipatory errors. There was also a main effect of Group (*F*(1, 50) = 6.543, *p* = 0.014, $\eta_{{\text{P}}}^{2}$ = 0.116; BF_incl_ = 3.81), a significant 2-way interaction between cue type and SOA (*F*(2, 100) = 14.60, *p* < 0.001, $\eta_{{\text{P}}}^{2}$ = 0.20, BF_incl_ = 126,608.33 and a significant interaction between cue type and Group (*F*(2, 100) = 4.75, *p* = 0.011, $\eta_{{\text{P}}}^{2}$ = 0.087, BF_incl_ = 1.25). The later 2-way interaction reflected the fact that anticipatory responding was similarly high for all cue types in PDs (Fingers versus Eyes: *t*(20) = 0.71, *p* = 0.487; Fingers versus Arrow: *t*(20) = 0.76, *p* = 0.46; Arrow versus Eyes: *t*(20) = 0.11, *p* = 0.91), whereas errors were greatest for the Finger and Arrow cues relative to Eye gaze cues in older controls (Fingers versus Eyes: *t*(30) = 5.04, *p* < 0.001; Fingers versus Arrow: *t*(30) = 3.71, *p* = 0.046; Arrow versus Eyes:* t*(20) = 2.07,* p* = 0.048) (Fig. 4).

#### Correlations with Parkinson’s disease severity and cognitive test scores

Pearson correlations were also carried out to investigate associations between Parkinson’s disease severity (UPDRS III scores) and cue congruency effects for each of the three cue types (Arrows, Eyes, Pointing) for the 3 dependent measures: SRTs, anticipatory errors and directional errors. None of the correlations showed a significant association between UPDRS and cue congruency effects at standard significance level (*α* = 0.05).

Correlations were carried out between the size of the eye movement cueing effects and scores on cognitive tests for the sub-set of patients completing the neuropsychological tests and for the MMSE for all patients. These showed correlations between MMSE scores and the congruency effect on SRTs for Arrow Cues (*ρ* = 0.553, *p* = 0.033) and the congruency effect on directional errors for Finger cues (*ρ* = − 0.439, *p* = 0.047), with all other correlations tested being statistically insignificant at standard significance level (*α* = 0.05).

#### Between experiment analysis

A series of 4-way ANOVAs with Task (Prosaccade, Antisaccade), Group (Patient, Healthy Control), cue type (Arrows, Eyes, Pointing) and cue congruency (congruent/incongruent) as factors, were used to compare the findings of Experiment 1 and 2 for each of the dependent measures. As only four of the patients and one of the controls took part in both Experiments, Task was entered as between subject factors alongside Group, with cue type and congruency treated as repeated measures factors.

This analysis confirmed the existence of Task by Group interactions for SRTs (*F*(1, 64) = 4.08, *p* = 0.048, $\eta_{{\text{P}}}^{2}$ = 0.06; BF_incl_ = 1.23), directional errors (*F*(1, 82) = 6.42, *p* = 0.013, $\eta_{{\text{P}}}^{2}$ = 0.073; BF_incl_ = 3.62) and anticipatory errors (*F*(1, 83) = 4.701, *p* = 0.033, $\eta_{{\text{P}}}^{2}$ = 0.054; BF_incl_ = 2.27), reflecting increased errors and SRTs for patients in the antisaccade task only. SRTs also showed a significant cue type by congruency interaction effect (*F*(2, 128) = 5.68, *p* = 0.004, $\eta_{{\text{P}}}^{2}$ = 0.081; BF_incl_ = 10.33), due to the larger cueing effects for the Pointing finger cues for both participant groups across both tasks. The analysis of anticipatory errors further showed a 4 way interaction between Task, Group, cue type and congruency (*F*(2, 166) = 3.046, *p* = 0.05, $\eta_{{\text{P}}}^{2}$ = 0.004; BF_incl_ = 1.46).

## General discussion

This study aimed to explore whether orienting in response to social directional cues might be affected in PD. Using an eye movement spatial cueing task, the effect of centrally presented arrows, eye gaze and finger pointing cues on SRTs and errors was measured in PDs and healthy controls. In contrast to the finding of normal SRTs and error rates in the prosaccade version of the task (Experiment 1), in Experiment 2 antisaccade directional errors and SRTs were significantly increased in PDs relative to controls. Some differences in the effect of the different types of cue images presented on each trial were also observed, with arrows and fingers generally showing stronger effects than eye gaze cues. Patients also differed significantly from controls in terms of the influence of the different cue types on the occurrence of anticipatory saccades, whereby controls made more anticipatory responses to finger cues, where anticipatory responding was equally high for all cue types in PDs (Fig. 4).

Although there is some inconsistency in the literature concerning whether antisaccade errors are increased in PD, the majority of studies report significantly increased directional errors in the task (Antoniades and Kennard 2015; Chan et al. 2005; Briand et al. 1999; Fukushima et al. 1994; although see Lueck et al. 1990; Rivaud et al. 2007). In our experience there is considerable heterogeneity between Parkinson's patients with respect to antisaccade errors, perhaps reflecting different neurological trajectories of disease progression, which may in part explain why some studies have not shown significant increases in errors at the group level in patients (Hodgson et al. 2019). The present results confirmed that as a group, given sufficient sample size, patients with mild to moderately severe symptoms have significantly increased directional errors in the antisaccade test as well as longer SRTs. Directional errors were also observed in the prosaccade task (Experiment 1), but when data from the two experiments were combined, a significant 2-way interactions between task and group for SRTs and errors confirmed a selective impairment for antisaccades relative to prosaccades.

Earlier studies have shown that antisaccade reaction times are shorter and error rates lower when eye gaze cues point towards the target location (Gregory and Hodgson 2012; Wolohon and Crawford 2012). This suggests that central cues affect antisaccades either via facilitation of covert attention towards the location in which they point, or due to participants adopting an “anti-orienting” set (Wolohon and Crawford 2012), whereby a saccade is programmed in the opposite direction to both central and peripheral cues. In the present study eye gaze cues produced a non-significant reverse congruency effect on SRTs, whereas finger pointing cues showed positive cue-congruency effects on SRTs (Fig. 3) and errors (Table 3). Directional errors were found to be increased when the finger pointed towards the peripheral target, consistent with the anti-orienting hypothesis. As with the findings for Experiment 1 this suggests that eye gaze cues may not produce strong cueing effects on SRTs in older adults. Other work has shown a reduction in the effect of gaze cues on eye movements in older relative to younger adults (Kuhn et al. 2015; De Roche et al. 2016). It is possible that this may reflect poorer vision and visual perceptual functioning in older adults rather than differences social or cognitive processing. However, studies that have controlled for visual acuity indicate that this is unlikely (Bailey et al. 2014), with one study of gaze cueing effects finding *increased* visual acuity in older relative to younger adults (Kuhn et al. 2015).

The rate of anticipatory errors (where a response was executed in the direction of the cue before or less than 80 ms after the target onset) were significantly increased in patients. There was also an interesting interaction effect for these errors between Group and cue type. Superficially the presence of this interaction might suggest that patients have a selective impairment in the processing of one or other of the different cue types. However, closer inspection of the interaction suggests a different explanation. Whereas control participants were particularly prone to making anticipatory saccades when a pointing finger image was presented compared to eye gaze or arrow cues, Parkinson’s patients appeared equally distractible under all three cue conditions (Fig. 4). This is consistent with the suggestion made elsewhere that PDs may “over respond” by generating actions to abstract stimuli which lack strongly established action affordance (Galpin et al. 2011). In the present situation, whereas healthy participants are more able to suppress the influence of arrow and eye gaze cues on saccade responding than they are for pointing finger cues, PDs are less able to suppress the distracting influence of all cue types on anticipatory saccade execution.

Rather than any selective impairment in processing of socially relevant cues or stimuli with an action affordance association (Poliakoff et al. 2007; Galpin et al. 2011), the findings of the two experiments taken together suggest that Parkinson’s patients suffer from a general disruption to the normal processing of visuospatial cues regardless of whether they are social or non-social in nature. We suggest that this is due to an inability to suppress the influence of directional cues on saccade programming and execution. In Experiment 1, healthy participants showed differences in the effect of cues at different SOAs for both prosaccades and antisaccades, suggesting that they can suppress the immediate influence of directional cues on saccade generation within 500 ms. Parkinson's patients did not show this interaction between congruency and SOA. Furthermore, they were found to be equally affected by all cue types in the antisaccade task, whereas stronger effects on anticipatory errors were observed for arrows and pointing fingers relative to eye gaze cues in healthy controls. The fact that the differences between patients and controls with respect to the effect of the directional cues were greater in the antisaccade task also supports the idea that they reflect difficulty in implementing response inhibition control, as the antisaccade task places additional demands on inhibitory control due to its requirement to suppress the stimulus elicited saccade towards the target stimulus.

This suggestion, that PDs have difficulty resolving the distracting influence of visuospatial cues, is also consistent with the wider literature on attention and cognitive functioning in Parkinson's. Patients with Parkinson's appear to be more susceptible to distracting stimuli in visual and memory-guided search tasks (Mannan et al. 2008; Hodgson et al. 2019); irrelevant stimuli during task switching, at least when in an unmedicated state (Cools et al. 2001); and the ability to suppress actions compatible with stimulus location in a “simon effect” task (Praamstra and Platt 2001). Difficulty in suppressing the influence of the cues on response selection mechanisms is also consistent with the greater influence of directional cues on saccades shown by patients in the spatial cueing task. This applies equally to social as well as non-social symbolic directional cues such as arrows, and the current study offers no support for the existence of dedicated brain circuits or modules for social processing as had been suggested elsewhere (Driver et al. 1999; Baron-Cohen 1994). Instead, top–down influences combine with stimulus elicited activity within prefrontal-striatal circuits to determine the influence of directional cues on response execution, irrespective of whether cues have social relevance or not. Some cues appear to exert more powerful influences than other (in the present study pointing finger cues showed particularly strong effects), but this is likely to reflect intrinsic properties of the cue image (e.g., physical asymmetry), or learned associations between cues and direction, rather than privileged status for sociobiological cues (Gregory et al. 2016).

A number of limitations of the present work can be identified. We acknowledge that participant and trial numbers used in the current study were low and as such the study may suffer from low statistical power. However, high inclusive Bayes factors values (BF_incl_ larger than 3) were observed for main effects of cue type, SOA and Group and interactions between SOA and cue type and congruency, suggesting that the data offer strong evidence in favour of models which include these effects, although higher order interactions (e.g., between Group and cue type for the antisaccade task) were inconclusive relative to alternative models (BF_incl_ between 0.33 and 3). The addition of “catch trials”, where a cue is presented but no target appears on a minority of trials would also have been useful to determine whether saccade responses were anticipatory or were driven by the onset of the target stimulus. It is likely that many directional errors are likely to have been responses executed in response to the cue even though they had longer response times than the most commonly used cut-off for deeming a saccade to be anticipatory (< 80 ms). Finally, the limited additional neuropsychological testing carried out in a sub-set of patients revealed a number of significant correlations with eye movement task measures (e.g., digit span and MMSE with magnitude of SRT cueing effects). This suggests that relatively simple tests of eye movements and spatial attention might be sensitive to onset of cognitive decline in patients. Application of comprehensive standardised cognitive tests across all participants would, therefore, have been informative for establishing whether attentional/oculomotor deficits in patients relate to the presence of cognitive impairments.

In summary, the results overall suggest that Parkinsons patients may be less able to suppress the influence of directional attentional cues on eye movement generation, but that this effect is not specific to cues of a sociobiological nature. Although the findings do not indicate any specific deficit in social attention in patients, general visuospatial attentional difficulties might translate into real world problems for patients when faced with situations in which eye movements and visual attention need to be directed within a busy environment, including commonly encountered social situations. This may be particularly challenging in situations where there are demands on executive and inhibitory control mechanisms, for example in a cluttered visual environment or when trying to pay attention to a task whilst having a conversation. Research using real world eye tracking in social situations may prove particularly informative in understanding how such deficits translate into real world problems for Parkinsons patients.
